# The role of peripheral immunity in ALS: a population‐based study

**DOI:** 10.1002/acn3.51853

**Published:** 2023-07-22

**Authors:** Maurizio Grassano, Umberto Manera, Fabiola De Marchi, Paolo Cugnasco, Enrico Matteoni, Margherita Daviddi, Luca Solero, Alessandro Bombaci, Francesca Palumbo, Rosario Vasta, Antonio Canosa, Paolina Salamone, Giuseppe Fuda, Federico Casale, Letizia Mazzini, Andrea Calvo, Cristina Moglia, Adriano Chiò

**Affiliations:** ^1^ ALS Centre, Department of Neuroscience “Rita Levi Montalcini” University of Torino Turin Italy; ^2^ SC Neurologia 1U AOU Città della Salute e della Scienza of Torino Turin Italy; ^3^ Department of Neurology and ALS Centre University of Piemonte Orientale, Maggiore della Carità Hospital Novara Italy

## Abstract

**Background:**

Systemic inflammation has been proposed as a relevant mechanism in amyotrophic lateral sclerosis (ALS). Still, comprehensive data on ALS patients' innate and adaptive immune responses and their effect on the clinical phenotype are lacking. Here, we investigate systemic immunity in a population‐based ALS cohort using readily available hematological indexes.

**Methods:**

We collected clinical data and the complete blood count (CBC) at diagnosis in ALS patients from the Piemonte and Valle d'Aosta Register for ALS (PARALS) from 2007 to 2019. Leukocytes populations, neutrophil‐to‐lymphocyte ratio (NLR), platelet‐to‐lymphocyte ratio (PLR), systemic‐immune‐inflammation index (SII), and lymphocyte‐to‐monocyte ratio (LMR) were derived from CBC. All variables were analyzed for association with clinical features in the entire cohort and then in sex‐ and age‐based subgroups.

**Results:**

Neutrophils (*P* = 0.001) and markers of increased innate immunity (NLR, *P* = 0.008 and SII, *P* = 0.006) were associated with a faster disease progression. Similarly, elevated innate immunity correlated with worse pulmonary function and shorter survival. The prognosis in women also correlated with low lymphocytes (*P* = 0.045) and a decreased LMR (*P* = 0.013). ALS patients with cognitive impairment exhibited lower monocytes (*P* = 0.0415).

**Conclusions and Relevance:**

The dysregulation of the systemic immune system plays a multifaceted role in ALS. More specifically, an elevated innate immune response is associated with faster progression and reduced survival. Conversely, ALS patients with cognitive impairment showed a reduction in monocyte count. Additionally, immune response varied according to sex and age, thus suggesting that involved immune pathways are patient specific. Further studies will help translate those findings into clinical practice or targeted treatments.

## Introduction

Neuroinflammation has been postulated to be a relevant mechanism in the neurodegenerative process in amyotrophic lateral sclerosis (ALS).^1^ Previous studies found that the frequency and activation of immune cell populations such as T cells, monocytes, and neutrophils have been associated with disease severity and rate of disease progression.^2^, ^3^, ^4^, ^5^ Innate immunity involves diverse types of cells of the myeloid lineage, including dendritic cells, monocytes, macrophages, polymorphonuclear cells, mast cells, innate lymphoid cells, and natural killer (NK) cells.^6^, ^7^ Adaptive immunity is characterized by two types of lymphocytes, T and B cells.^6^ The most easily and generalizable method to quantify peripheral immunity is blood cell counting of differential leukocytes. Furthermore, derived ratios including neutrophil‐to‐lymphocyte ratio (NLR), platelet‐to‐lymphocyte ratio (PLR), systemic immune‐inflammation index (SII),^8^ and lymphocyte‐to‐monocyte ratio (LMR) were thought to even better reflect the strength of peripheral immunity.^9^ There have been studies suggesting that these ratios could serve as systemic biomarkers of ALS prognosis,^10^, ^11^, ^12^ but whether they are correlated with other specific features of ALS phenotype, including lung function, is still unknown. Additionally, the potential effect of peripheral immunity on non‐motor symptoms, such as cognitive impairment, has not been explored so far. Because systemic inflammation is a complex and a multifactorial process, however, a better interpretation of the immune profile in ALS patients requires the simultaneous study of different biomarkers.

Here, we describe the systemic immune role in ALS patients by analyzing complete blood count (CBC)‐derived parameters in a large population‐based cohort. A better understanding of the systemic immune response in the context of ALS and frontotemporal dementia (FTD) is not only important to unravel disease mechanism, but potentially to serve as biomarker of disease activity.

## Materials and Methods

### Study population

All patients with ALS in the Piemonte and Valle d'Aosta regions of Italy (*n* = 1784) identified through the Piemonte and Valle d'Aosta Register for ALS and diagnosed between January 1, 2007, and December 31, 2019, were eligible for enrollment in the study. All patients met the revised El Escorial diagnostic criteria for definite and probable laboratory‐supported ALS. A complete clinical history, including previous disease (especially history of cancer and inflammatory disease) and smoking habit (current, past, or never smoker), was collected for each patient. We restricted analyses to available blood count data within 3 months of ALS diagnosis. In addition, participants with morbidities that could influence leukocyte differential counts, including malignant neoplasms, disease of the blood and blood‐forming organs, autoimmune disease, and chronic inflammatory diseases, were excluded. To avoid the confounding effect of acute infection, blood tests executed during suspected infections were detected and excluded. Additionally, we reviewed clinical records and removed patients undergoing pharmacological treatment at the time of diagnosis that could alter leucocytes populations.

### Peripheral immunity

Patients underwent hematological examinations as part of the diagnostic workup. Peripheral blood sample was drawn after overnight fasting, and the complete blood count was quantified in the Department of Biochimica Clinica, AOU Città della Salute e della Scienza of Torino, Turin, Italy, and the Department of Biochimica Clinica, Maggiore della Carità Hospital, Novara, Italy. We extracted baseline count data of neutrophils, monocytes, platelets, and lymphocytes. Further we calculated four ratios based on peripheral blood cell counts including NLR (neutrophils/ lymphocytes), PLR (platelets/lymphocytes), SII (neutrophils*platelets/lymphocytes), and LMR (lymphocytes/monocytes). The increased level of the neutrophils, monocytes, NLR, PLR, and SII reflects the relatively higher peripheral innate immunity, while the increased level of the lymphocytes and LMR reflects the relatively higher peripheral adaptive immunity.

### Clinical features

Disease severity was assessed with the Amyotrophic Lateral Sclerosis Functional Rating Scale–Revised scale (ALSFRS‐R). In addition, the decline rate for ALSFRS‐R score and its four sub‐scores (bulbar, fine motor, gross motor, and respiratory) was calculated as the monthly number of points lost from symptom onset to the time of diagnosis. Pulmonary function tests, including forced vital capacity (FVC), were performed at diagnosis. Respiratory dysfunction was defined as FVC < 75% of the predicted value. Body mass index (BMI) was calculated as weight in kilograms divided by height in meters squared, and its decline was calculated as monthly the difference in premorbid BMI and BMI at diagnosis, divided by months from symptom onset to diagnosis. Patients' cognitive status was classified as previously described according to the revised ALS‐FTD Consensus Criteria into five categories: ALS with normal cognition (ALS‐CN), ALS with behavioral impairment (ALS‐Bi), ALS with cognitive impairment (ALS‐Ci), ALS with cognitive and behavioral impairment (ALS‐CBi), and ALS with FTD (ALS‐FTD).

King's staging, that categorizes ALS based on the spread of motor symptoms in three body regions (bulbar, upper limbs, and lower limbs) and the use of non‐invasive ventilation (NIV) and enteral nutrition, was calculated from ALSFRS‐R items as follows: bulbar region is determined by a decrease in points related to speech, salivation, or swallowing (items 1, 2, 3); the upper limb region is considered involved if there is a decrease in points for handwriting or ability to handle utensils (items 4, 5); the lower limb region is considered involved if there is a decrease in points for walking (items 6); the presence of gastrostomy is indicated by item 5B and NIV usage is indicated by scoring 0 points in item 10 or less than 4 points in item 12. Patients are classified into four stages: 1 (one region involved), 2 (two regions involved), 3 (three regions involved), and 4 (requiring gastrostomy or NIV). Survival was calculated from onset to death or censoring date (December 31, 2019) using the Kaplan–Meier method. Patients with tracheostomy were coded as deceased on the date of the procedure.

### Statistical analyses

Baseline characteristics of participants were analyzed as mean and standard deviation (SD) for continuous variables and as numbers and percentages for categorical variables. Given the high interpersonal variability of baseline FVC values, we elected to assess respiratory function as a dichotomous variable (FVC < 75% of predicted value). Prior to statistical analysis, peripheral immunity cell counts were log‐transformed and standardized to *Z* scores for comparison of effect sizes between exposures (*Z* = (value−mean)/SD) such that the hazard ratio (HR) represents the predicted effect of per SD increment of the peripheral immunity markers. In the primary analysis, we performed the models unadjusted, then adjusted for age, sex, smoking history, presence of bulbar dysfunction (score <4 in either item 1, 2, or 3 of the ALSFRS‐R scale) and body mass index (BMI). We performed a Linear Mixed Effect regression on continuous and binary variables with random effect to account for potential inter‐center variability. Association between peripheral immunity markers and survival was examined with multivariable Cox proportional hazard regression models. The *P* values were further adjusted to control the false discovery rate at 5% using the Benjamini–Hochberg procedure (labeled as *Q* values). For each biomarker, we also performed the analysis excluding patients with extreme values (>mean ± 2SD) in at least one of the other exposures, thus correcting for potential correlation across different biomarkers. In stratified analyses, we estimated differences for each exposure across different age groups (<65, 65–75, >75 years) and sex. We elected to use age cutoffs to favor clinical interpretation and future replication in different cohorts. These age cutoffs were arbitrarily chosen based on the characteristics of the population (Table S1) and to obtain comparable groups. Finally, inflammatory markers, age, and sex were modeled with an interacting parameter to evaluate the potential interplay among those three factors. Statistical analysis was performed in R v4.0.

### Ethical approval

The study design was approved by the institutional ethical committees of Azienda Ospedaliera Universitaria Città della Salute e della Scienza, Torino (#355732), and Azienda Ospedaliera Universitaria Maggiore della Carità, Novara (#6739A4). All patients provided written informed consent.

## Results

### Population characteristics

Among the 1784 patients included in the PARALS Registry in the study period, 1452 (81.5%) subjects were eligible for analysis. The final cohort consisted of 653 (44.9%) females and 799 (55.1%) males. Median age at disease onset was 69.5 years (IQR: 61.4–75.7); bulbar onset was observed in 489 (33.7%) cases. Descriptive statistics of the participants are summarized in Table S1. Patients were excluded if blood count data were not collected within 3 months of ALS diagnosis (*n* = 77); blood tests were performed because infection was suspected (*n* = 26); treatment with leucocytes‐altering drugs was undergoing (*n* = 60); patients had a known history of malignancy (*n* = 110), hematological disorders, or autoimmune disease (*n* = 59). Patients excluded from the study did not differ in term of demographic or other clinically relevant variables (Table S1). Blood count derived data are reported in Table S2. Data according to sex, age groups, and site of disease onset are summarized in Tables S2–S5; potential confounding effect of the presence of bulbar symptoms at the time of evaluation or smoking habits is considered in Tables S6–S8.

### Baseline

**Figure 1:**
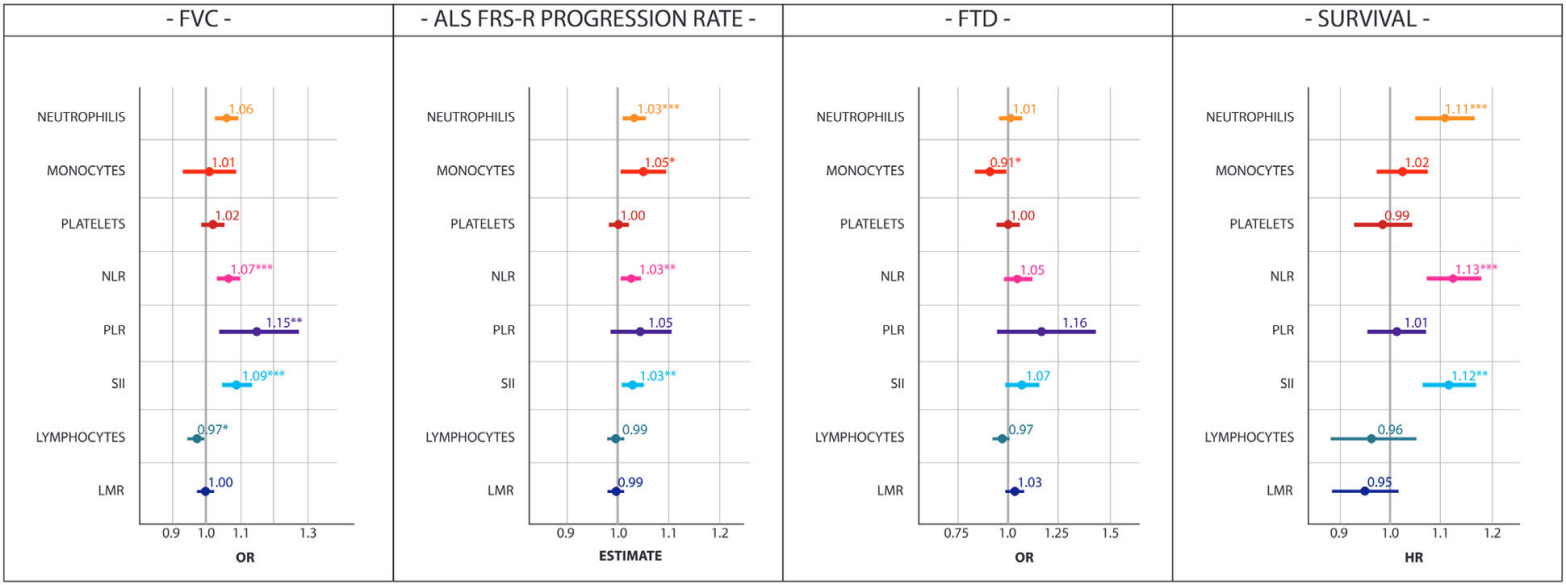
Overall association of peripheral immune markers with FVC < 75% of predicted value, ALSFRS‐R progression rate, presence of FTD, and survival. *β* estimates from linear regression; OR odds ratio from logistic regression, HR hazard ratio from Cox proportional hazard regression, 97.5% CI, 97.55% confidence interval; LMR, lymphocyte‐to‐monocyte ratio; NLR, neutrophil‐to‐lymphocyte ratio; PLR, platelet‐to‐lymphocyte ratio; SII, systemic inflammatory index.

After adjustment for age, sex, presence of bulbar involvement, smoking habits, and BMI, per SD increment of neutrophils, the main components of innate immunity, was associated with higher rates of disease progression (*β*: 1.03, 97.5% CI: 1.01–1.05, *P* = 0.001) (Fig. 1 and Table S8). Similar findings were observed in higher ratios of the markers reflecting innate immunity over adaptive immunity, including NLR (per SD increment *β*: 1.03, 97.5% CI: 1.01–1.05, *P* = 0.008) and SII (per SD increment *β*: 1.03, 97.5% CI: 1.01–1.05, *P* = 0.006). No significant effect was found for platelets and PLR on progression rates. The level of lymphocytes, the core adaptive immunity components, was not associated with progression rates. Results were not significant also for LMR. Patients with a faster disease progression also exhibited an increase in peripheral monocytes (*β*: 1.05, 97.5% CI: 1–1.09, *P* = 0.041). Importantly, while smoking status moderately affected the neutrophil count (Table S6), multivariate analysis confirmed that the association of innate immunity with disease progression was independent from smoking status. Additionally, no systemic inflammation signature was affected by bulbar dysfunction (Table S7).

As for pulmonary function, a consistent association with respiratory failure (here defined as FVC% < 75% of the predicted value) was found in innate immunity, as presented for neutrophils (per SD increment OR: 1.06, 97.5% CI: 1.03–1.09, *P* < 0.001) NLR (per SD increment OR: 1.07, 97.5% CI: 1.03–1.10, *P* < 0.001), PLR (per SD increment OR: 1.15, 97.5% CI: 1.04–1.28, *P* = 0.007), and SII (per SD increment OR: 1.09, 97.5% CI: 1.05–1.14, *P* < 0.001), respectively (Fig. 1). Conversely, an association of adaptive immunity with higher FVC values was observed in adjusted models for lower lymphocyte levels (OR: 0.97, 97.5% CI: 0.95–0.99, *P* = 0.030).

Unsurprisingly, given the association with disease progression rates and respiratory function, innate immunity markers also correlated with a shorter disease survival (Fig. 1). No direct association was found regarding altered adaptive/regulatory pathways in ALS subjects with a shorter survival.

Overall, an increase in innate immune response was observed in patients with a more advanced disease stage at diagnosis (Table S9): Indeed, higher disease stages were associated with increase in neutrophil levels (per SD increment OR: 0.06, 97.5% CI: 0.01–0.11, *P* = 0.010), NLR (per SD increment OR: 0.07, 97.5% CI: 0.02–0.12, *P* = 0.006), PLR (per SD increment OR: 0.24, 97.5% CI: 0.09–0.40, *P* = 0.002), and SII (per SD increment OR: 0.11, 97.5% CI: 0.05–0.16, *P* < 0.001).

Concerning FTD and cognitive symptoms incidence, higher levels of monocytes were related to decreased risk of FTD (per SD increment OR: 0.92, 97.5% CI: 0.86–0.99, *P* = 0.047) (Table S9) and cognitive impairment (per SD increment OR: 0.84, 97.5% CI: 0.73–0.97, *P* = 0.017) (Fig. 1).

### Peripheral immunity and ALS phenotypes across different ages and sex

To understand whether the observed relationship between peripheral immunity and ALS clinical features was influenced by age or sex, both known modifiers of the immune response, we performed a series of subgroup analyses.

**Figure 2:**
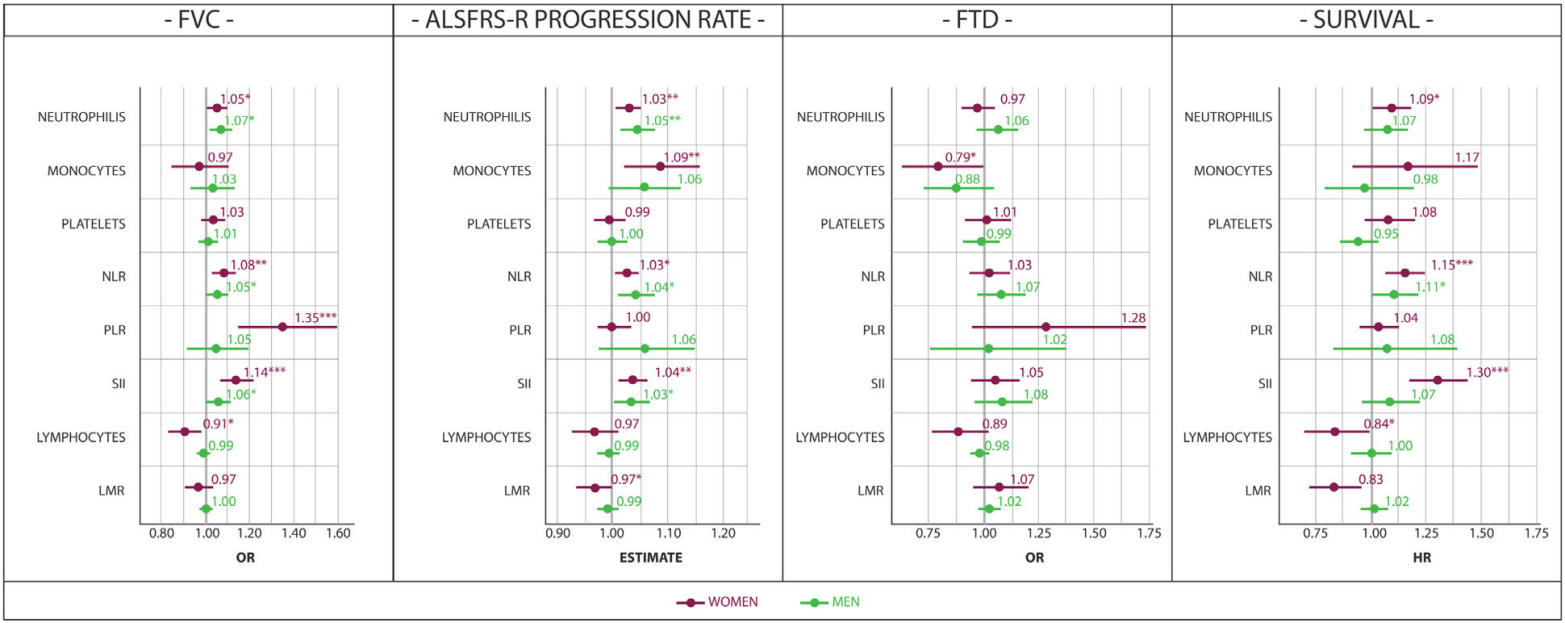
Sex‐stratified association of peripheral immune markers with FVC < 75% of predicted value, ALSFRS‐R progression rate, presence of FTD and survival. *β* estimates from linear regression; OR odds ratio from logistic regression, HR hazard ratio from Cox proportional hazard regression, 97.5% CI, 97.55% confidence interval; LMR, lymphocyte‐to‐monocyte ratio; NLR, neutrophil‐to‐lymphocyte ratio; PLR, platelet‐to‐lymphocyte ratio; SII, systemic inflammatory index. Male, *n* = 799; Female, *n* = 653.

Firstly, we set apart the population by sex (Table S10). The effect of innate immunity was explicit for disease progression and survival either in males or females (Fig. 2). Significant differences between the two sexes emerged regarding the mediator of immunity, as evidence of a detrimental effect of low lymphocyte count on prognosis was found in females (per SD increment Cox‐HR: 0.84, 97.5% CI: 0.70–0.99, *P* = 0.045) but not in males. Comparable results were obtained for LMR (per SD increment Cox‐HR: 0.84, 97.5% CI: 0.72–0.96, *P* = 0.013). By contrast, innate immunity marker that accounts for an increased neutrophil count was correlated with disease progression and prognosis in both sexes. Interestingly, only males exhibited an increase in all markers of innate immune response for higher disease stage at the time of diagnosis (Table S10).

Higher monocyte count was also proven to slightly decrease the incidence of cognitive deficits only in females (per SD increment OR: 0.79, 97.5% CI: 0.63–0.99, *P* = 0.046) (Fig. 2). Curiously, the opposite trend was observed in progression rate, where we observed a more severe disease progression when monocyte levels increased (per SD increment *β*: 1.09, 97.5% CI: 1.02–1.16, *P* = 0.009) (Fig. 2).

**Figure 3:**
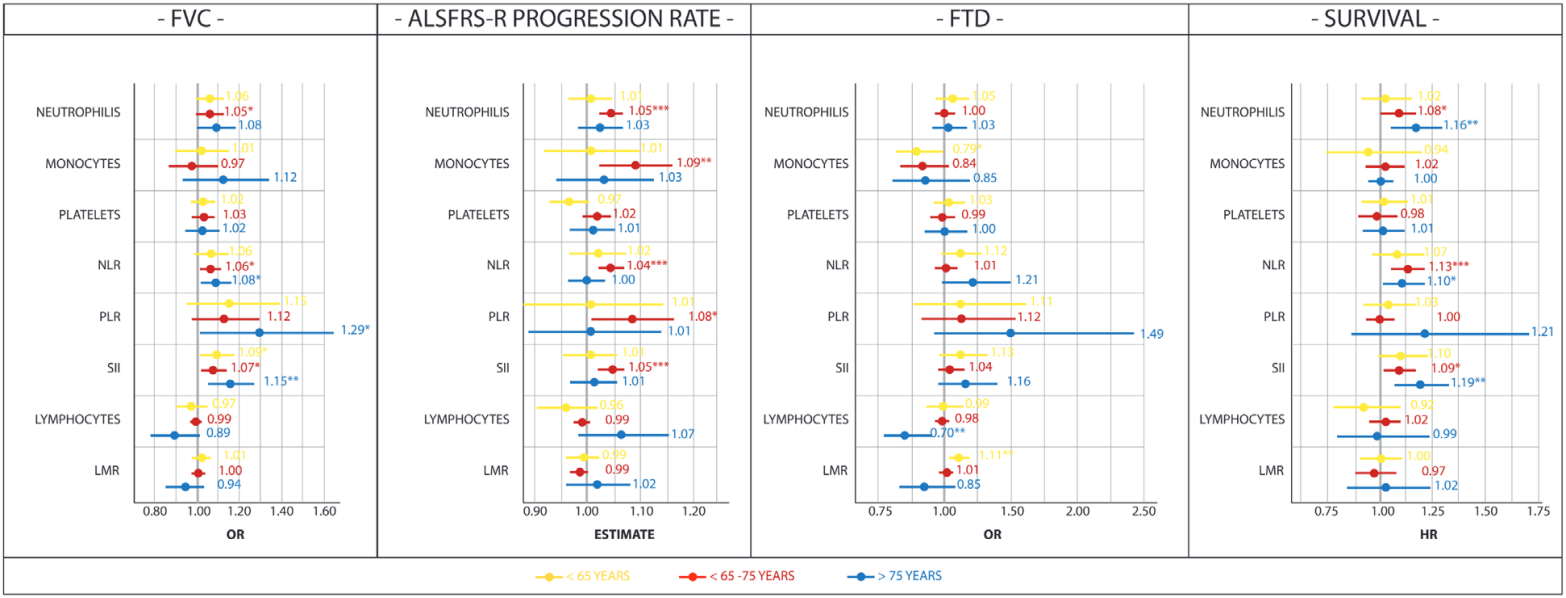
Age‐stratified association of peripheral immune markers with FVC < 75% of predicted value, ALSFRS‐R progression rate, presence of FTD and survival. *β* estimates from linear regression; OR odds ratio from logistic regression, HR hazard ratio from Cox proportional hazard regression, 97.5% CI, 97.55% confidence interval; LMR, lymphocyte‐to‐monocyte ratio; NLR, neutrophil‐to‐lymphocyte ratio; PLR, platelet‐to‐lymphocyte ratio; SII, systemic inflammatory index. Age < 65 years, *n* = 497; age 65–75 years, *n* = 548; age >75 years, *n* = 406.

Subsequently, we divided the included participants into three groups by age (<65, 65–75, >75 years). In participants younger than 65 years (Fig. 3, Table S11), only one innate immunity marker (the SII) increased with worsening respiratory function, but not with disease progression or survival (Fig. 3A,B,D). Low‐monocytes (OR: 0.79, 97.5% CI: 0.63–0.99, *P* = 0.041) and therefore an increased LMR (OR: 1.11, 97.5% CI: 1.03–1.19, *P* = 0.006) were found to be associated with cognitive impairment in young‐onset ALS cases. Surprisingly, lymphocytes (OR: 0.70, 97.5% CI: 0.54–0.90, *P* = 0.006) are decreased in older patients (>75 years) with cognitive impairment (Fig. 3). In addition to reduced lymphocyte, we also observed increased NLR (OR: 1.15, 97.5% CI: 1.02–1.30, *P* = 0.021) and SII (OR: 1.13, 97.5% CI: 1.01–1.26, *P* = 0.030) in older ALS patients with an overlapping FTD (Table S11). When it moves to 65–75 years and over 75 years, significant detrimental roles were found for the increase of all innate immunity markers accounting for neutrophil levels (neutrophils, NLR and SII) in disease progression and survival (Fig. 3). The relevance of age on determining the role of systemic immune response in ALS was further highlighted by the absence of associations between innate immune responses and disease stages in both younger and older patients. However, it was notably observed that this association was evident among patients with intermediate age (Table S11).

We also find that the different immunological signatures between men and women are not static, as we observed age‐by‐sex interaction for the clinical traits we investigated. Innate immunity parameters correlated with lung function and survival, such as neutrophils, NLR and SII levels, do not show an age‐based difference when estimated in men, but manifested an association that increased with age when estimated in women (Table S12). Those results suggest that the effect of innate immunity is less pronounced in younger patients, with important sex‐based differences.

## Discussion

**Figure 4:**
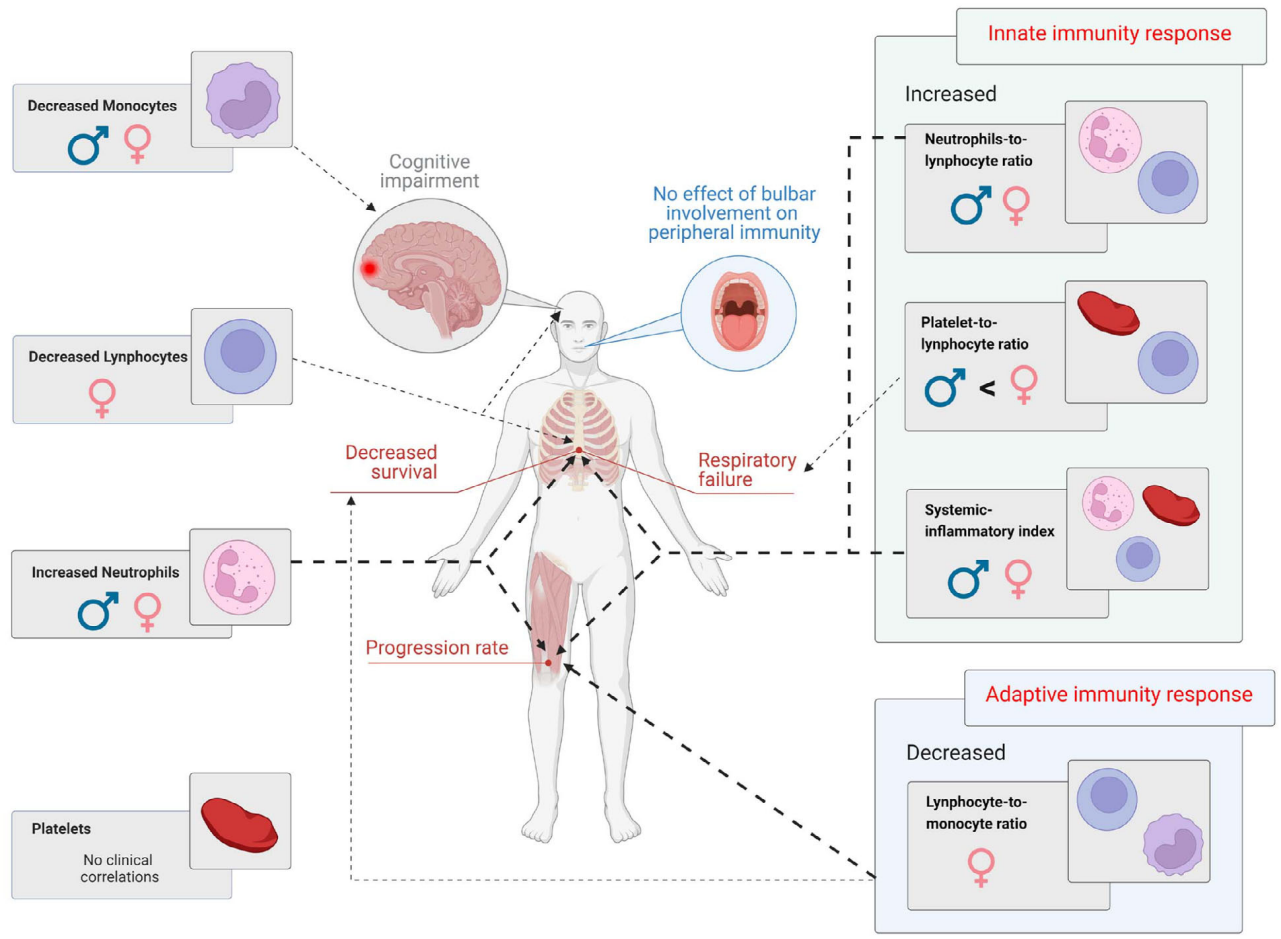
Graphical summary of study results. Baseline neutrophil count and increased markers of innate immune response in the peripheral blood are associated with a shorter survival time via multiple potential mechanisms, such as a more rapid motor disease progression and a lower respiratory function. In addition, a reduced monocyte count was observed in patients with more severe cognitive impairment. Lower lymphocyte levels and markers of adaptive immune response correlated with shorter survival, with lower lymphocytes also linked to cognitive decline. However, these effects of lymphocytes and adaptive immunity were only observed in female patients.

In this study, we comprehensively evaluated multiple CBC‐derived inflammatory indexes, including novel candidate markers, in a large population‐based ALS cohort. Our analysis supports the contribution of various immune cell types to different features of disease phenotype: a graphic summary of our results can be found in Figure 4. Our results reinforced that the peripheral immune system plays a significant role in the progression of ALS. These data add to the growing evidence suggesting that dysregulation of systemic immunity is a common feature among individuals with ALS; however, here we demonstrate that the role of peripheral immune response is multifaceted and varies according to the patient's characteristics (Fig. 4). Even though we could not comprehensively consider all cellular and molecular pathways underlying central and peripheral inflammation in ALS, we demonstrated that different inflammatory mechanisms act in ALS patients, perhaps also simultaneously. While our results need confirmation in other cohorts, it could be postulated that the evaluation of these readily available and cost‐effective peripheral biomarkers could assist clinicians in patients' management and could also be pivotal in population stratification or in the identification of subjects with a likely immune dysregulation that could be responders to a targeted treatment.

We found that elevated innate immune inflammatory status, reflected by increasing peripheral neutrophils and a higher ratio of neutrophils to lymphocytes, is associated with a more advanced disease stage at the time of diagnosis (Table S13), a reduced survival and faster disease progression. These data are consistent with previous studies that have linked neutrophil levels to disease progression and NLR with a higher mortality.^5^, ^10^, ^11^ The associations of inflammatory response on disease progression, pulmonary function, and prognosis are independent of the presence of smoking habit or bulbar dysfunction at diagnosis. In this regard, it is noteworthy that higher levels of all innate immune response markers are not associated with involvement of bulbar function, because it excludes potential confounding factors such as silent infection (i.e., pneumonia resulting from silent aspiration) that are common complications in patients with bulbar symptoms and which may trigger the inflammatory response. We also observed that other inflammatory biomarkers, namely decreased absolute lymphocyte count, higher monocyte count, and their increased ratio (lymphocyte‐to‐monocyte), are linked to disease severity and worse prognosis in ALS patients, especially women. This correlation between a reduction of total lymphocytes and a shorter survival showed that the adaptive immune response is also a key player in the systemic response in ALS cases: unfortunately, given the nature of our study, we are unable to detect any changes in lymphocytes subpopulations that have been extensively described in ALS.^2^, ^3^, ^5^, ^13^, ^14^, ^15^, ^16^

Our overall and stratified data demonstrated that an increased innate immune system at diagnosis is associated with a more aggressive disease course and confirms that neutrophils are protagonists of inflammatory response in ALS across all age groups and sex. However, according to our results, peripheral immune involvement in ALS is multifaceted and different mechanisms and leukocytes population are involved. Consequently, a single marker is not sufficient to capture the overall peripheral immune dysregulation and inflammatory status in ALS, and the evaluation of multiple markers is necessary to disentangle the complex underlying mechanism involved in ALS pathogenesis and progression.

Another novel finding from our study was the inverse association between monocyte levels and the degree of cognitive impairment in patients with ALS. Patients with greater cognitive impairment showed a reduction in all monocyte subpopulations. Our study could not unravel whether these changes are contributing to the ALS‐FTD spectrum or a result of it. However, there are several reports of increased CNS infiltration of monocytes in ALS patients, and prior studies have speculated that a reduction in peripheral blood monocytes could be due to the recruitment and infiltration of these cells into theCNS.^3^, ^17^ Moreover, deregulation of the different populations of peripheral blood monocytes and their subsequent entry into the CNS may contribute to the neurodegenerative process or have a modifying role in the context of ALS.^18^

In this context, it is worth noting that monocytes manifested an opposite trend regarding motor progression (the faster the functional decline, the higher the level of circulating monocytes): This result questions whether we are observing two distinct phases of a single inflammatory response or rather the activation of different pathways. Although it remains unclear whether monocytes play a primary or secondary role in neurodegeneration, our findings point to a specific immune response in the subset of ALS patients with overlapping cognitive impairment or FTD. While further evidence is necessary, our data confirm that monocytes may represent an attractive target to study disease‐associated neuroinflammatory processes.

An effect on the risk of cognitive impairment was detected for adapted immunity: among older ALS patients (>75 years at diagnosis), lymphocyte count was decreased; younger ALS cases (<65 years at diagnosis) exhibited instead lower LMR levels, although this effect might be driven by the reduction in peripheral monocytes. Conversely, despite their correlation with motor progression in the entire cohort, innate immunity markers were associated with FTD‐like features only in late‐onset ALS patients. However, this correlation might reflect the changes in the lymphocyte populations.

Our data also clearly demonstrated that the immune response changes with patients' age: While we could not determine whether immune mechanisms contribute to the age‐related differences of ALS phenotypes, immunosenescence should carefully be considered when analyzing the role of immunity in the disease.

Finally, our findings suggest that the mechanism of immune response in ALS varies according to sex. When the analysis was stratified by sex, we found that while the effect of neutrophil levels remained consistent, low peripheral lymphocytes were associated with respiratory dysfunction and shorter survival only in females. Conversely, we observed a higher innate immune response in the more advanced disease stages exclusively in the male ALS population. The cause for this discrepancy is currently not clear, although sex‐based differences in peripheral immunity in ALS have been reported before.^19^ While further evidence is required to ponder whether sex‐based immunological differences contribute to the variation in the clinical manifestation that characterizes female and male ALS subjects,^20^ our results emphasized that sex is a variable that should be considered when studying phenotypes and biomarkers in motor neuron disorders.

Taken together, the results of this study described the age‐ and sex‐based effect of a multifaceted immune response on motor, cognitive, and respiratory symptoms of ALS patients.

Our study has some limitations. First, as already mentioned, we are unable to capture the effect of lymphocytes subpopulations. Notably, the compositional and functional status of T cells in blood and cerebrospinal fluid (CSF) in ALS patients was recently associated with both disease progression rates and survival^21^: Increased CD8^+^ T‐cell counts in the peripheral blood have already been linked to rapid disease progression,^5^, ^22^ while neuroprotective effects have been postulated for regulatory T cells and natural killer (NK) cells.^22^, ^23^, ^24^ Similarly, monocyte subpopulations variably contributed to the disease: While CD16^+^ monocytes have a potential protective role,^18^ CD16^−^ monocytes could actively participate to neurodegeneration and disease progression.^25^ However, considering the promising findings that have emerged from our analysis, there is reason to anticipate that the assessment of lymphocyte subpopulations could be incorporated into the evaluation of ALS patients.

Since we converted values from cell counts and ratio to z‐score, other efforts will be required to find clinically relevant and consistent cutoffs that could establish those indexes as reliable biomarkers in clinical practice. Furthermore, because of the retrospective nature of our cohort, the present study is not designed to answer whether targeting the peripheral immune system with therapeutic interventions could be beneficial for ALS patients.

Indeed, whether the observed alteration in systemic immunity is a cause or a consequence of disease progression and phenotypic variability in ALS is still unclear. For instance, neutrophils have several putative roles in ALS which could also differ according to the disease stage: Most prominently, neutrophils may either have a pro‐inflammatory role in driving neurodegeneration and promoting the breakdown of the brain–spinal cord barrier,^26^ or they could be involved in neuronal repair and increment in response to CNS damage. In this regard, it is noteworthy that the time from disease onset to diagnosis and blood collection did not influence leucocyte counts or inflammatory parameters in our cohort (Table S8): This should prompt questions about the dynamic of the inflammatory response during ALS course and whether repeated assessment could be necessary over time to identify to fully capture the complexity of immune response in ALS and its relationship with disease progression. Similarly, our data may reflect the dynamic alteration of peripheral immunity during the disease course, with mechanisms of innate immunity activation prevailing in later disease stages.

Additionally, a dysregulated immune response may also indirectly contribute to increased mortality in ALS through its correlation with respiratory function: Chronic inflammation is known to promote a decline in lung function, including forced vital capacity (FVC).^27^ In this regard, markers of inflammatory status like the neutrophil‐to‐lymphocyte ratio may reflect the adverse respiratory associations of exogenous exposures with lung function, like smoking and other nonsmoking exposures with respiratory outcomes (e.g., air pollution).^28^

While smoking habits did not significantly influence the inflammatory status in our cohort, it cannot be excluded that immune system functioning acts as a mediator of one of the several other environmental factors (nutrition status, pollutant exposures, physical activity, and the composition of microbiome among others) that are supposed to contribute to ALS.

This study validated the existence of peripheral immune abnormalities in ALS patients and explored the association between peripheral immunity and the clinical characteristics of ALS. We demonstrated that at diagnosis ALS patients with an increased innate immune activity had a faster progression of motor symptoms, a more advanced disease stage, a higher burden of cognitive impairment and reduced survival times. Different peripheral immune cells may specifically contribute to the multifaceted mechanisms and clinical features in ALS, as observed with monocytes and cognitive deficits. Finally, immune response in ALS may be sex‐specific, as adaptive immune response seems to play a more prominent role in females rather than in male ALS subjects. In conclusion, ALS is a disorder with extensive systemic pro‐inflammatory responses: Further studies are required to translate our findings into novel clinical markers and potential therapeutic targets.

## Author Contributions

MG, UM, ACalvo, CM, and AChiò contributed to the conception and design of the study; MG, UM, FDM, PC, EM, MD, LS, AB, FP, RV, ACanosa, PS, GF, FC, LM, ACalvo, CM, and AChiò contributed to the acquisition and analysis of data; MG, UM, FDM, LM, ACalvo, CM, and AChiò contributed to drafting the text or preparing the figures.

## Conflict of Interest

Maurizio Grassano, Umberto Manera, Fabiola De Marchi, Paolo Cugnasco, Enrico Matteoni, Margherita Daviddi, Luca Solero, Alessandro Bombaci, Francesca Palumbo, Rosario Vasta, Antonio Canosa, Paolina Salamone, Giuseppe Fuda, Federico Casale, Letizia Mazzini, Cristina Moglia report nothing to disclose. Andrea Calvo has received a research grant from Cytokinetics. Adriano Chiò serves on scientific advisory boards for Mitsubishi Tanabe, Roche, Denali Pharma, Cytokinetics, and Amylyx.

## Supporting information


**Table S1.** Descriptive statistics of the cohort.
**Table S2.** Summary of complete blood count (CBC) values and derived inflammatory markers according to sex.
**Table S3.** Summary of complete blood count (CBC) values and derived inflammatory markers according to site of disease onset.
**Table S4.** Summary of complete blood count (CBC) values and derived inflammatory markers according to age groups.
**Table S5.** Summary of complete blood count (CBC) values and derived inflammatory markers according to sex and age groups.
**Table S6.** Influence of smoking status on CBC data and inflammatory markers.
**Table S7.** Influence of bulbar symptoms on CBC data and inflammatory markers.
**Table S8.** Influence of the time from symptoms onset to blood sample collection on CBC data and inflammatory markers in the whole cohort (A), according to sex (B) and age groups (<60, 60–70, >70 years) (C).
**Table S9.** Summary of multivariate analysis of immune and adaptive inflammation markers in the whole cohort.
**Table S10.** Summary of multivariate analysis of immune and adaptive inflammation markers according to sex.
**Table S11.** Summary of multivariate analysis of immune and adaptive inflammation markers according to age group (<60, 60–70, >70 years).
**Table S12.** Interaction analysis of inflammatory markers, age, and sex.
**Table S13.** Summary of sensitivity analysis performed on ALSFRS‐R score at diagnosis in the whole cohort (A), according to sex (B) and age groups (<60, 60–70, >70 years) (C).

## Data Availability

Data are available for interested researchers upon reasonable request to the corresponding author.
