# Supplementary material for: The role of peripheral immunity in ALS: a population‐based study
Source: Ann Clin Transl Neurol. 2023 Jul 22;10(9):1623–32. doi: 10.1002/acn3.51853 (PMC10502618; doi:10.1002/acn3.51853)
Supplement: Supplementary file 1 — Table S1. Descriptive statistics of the cohort. Table S2. Summary of complete blood count (CBC) values and derived inflammatory markers according to sex. Table S3. Summary of complete blood count (CBC) values and derived inflammatory markers according to site of disease onset. Table S4. Summary of complete blood count (CBC) values and derived inflammatory markers according to age groups. Table S5. Summary of complete blood count (CBC) values and derived inflammatory markers according to sex and age groups. Table S6. Influence of smoking status on CBC data and inflammatory markers. Table S7. Influence of bulbar symptoms on CBC data and inflammatory markers. Table S8. Influence of the time from symptoms onset to blood sample collection on CBC data and inflammatory markers in the whole cohort (A), according to sex (B) and age groups (<60, 60–70, >70 years) (C). Table S9. Summary of multivariate analysis of immune and adaptive inflammation markers in the whole cohort. Table S10. Summary of multivariate analysis of immune and adaptive inflammation markers according to sex. Table S11. Summary of multivariate analysis of immune and adaptive inflammation markers according to age group (<60, 60–70, >70 years). Table S12. Interaction analysis of inflammatory markers, age, and sex. Table S13. Summary of sensitivity analysis performed on ALSFRS‐R score at diagnosis in the whole cohort (A), according to sex (B) and age groups (<60, 60–70, >70 years) (C). [file ACN3-10-1623-s001.docx]

**Supplementary Table 1. Descriptive statistics of the cohort.**

|  | **All patients (n=333)** | **Patient included in the study (n=1451)** |  |
| --- | --- | --- | --- |
|  | ***Median (IQR)*** | ***Median (IQR)*** | ***Mann-Whitney p-value*** |
| **Age at diagnosis** (years) | 70.1 (61.0-77.5) | 69.5 (61.4-75.7) | 0.322 |
| **Weight loss** (onset-diagnosis, kilograms) | 3.1 (0.0-9.0) | 3.0 (0.0-7.0) | 0.518 |
| **FVC** (%) | 86.0 (65.0-104.0) | 89.0 (70.0-103.0) | 0.258 |
| **ALSFRS-R RATE** (points lost/month) | 0.73 (0.40-1.50) | 0.66 (0.33-1.33) | 0.125 |
| **Overall survival** (months from diagnosis) | 18.8 (8.4-32.1) | 19.1 (9.0-35.1) | 0.230 |
|  | ***n (%)*** | ***n (%)*** | ***Chi-squared p-value*** |
| **Sex** |  |  |  |
| Male | 182 (54.7%) | 799 (55.1%) | 0.891 |
| Female | 151 (45.3%) | 652 (44.9%) |  |
| **Type of onset** |  |  |  |
| Bulbar onset | 109 (32.8%) | 489 (33.7%) | 0.736 |
| Spinal onset | 224 (67.2%) | 962 (66.3%) |  |

**Supplementary Table 2. Summary of complete blood count (CBC) values and derived inflammatory markers according to sex. P-values from logistic regression model.**

|  | **Total** (median, (IQR)) | **Sex** (median, (IQR)) | |  |
| --- | --- | --- | --- | --- |
|  | (n=1452) | **Male (n=799)** | **Female (n=653)** | **p-value** |
| **WBC** | 6.28 (2.34) | 6.35 (2.31) | 6.05 (2.34) | 0.027 |
| **Neutrophils** | 3.77 (1.96) | 3.77 (1.87) | 3.57 (1.93) | 0.077 |
| **Lymphocytes** | 1.72 (0.8) | 1.72 (0.76) | 1.73 (0.82) | 0.545 |
| **Monocytes** | 0.48 (0.23) | 0.49 (0.22) | 0.44 (0.23) | <0.0001 |
| **Platelets** | 229 (82) | 212 (80) | 243 (82.2) | <0.0001 |
| **NLR** | 2.14 (1.47) | 2.16 (1.43) | 2 (1.48) | 0.284 |
| **PLR** | 135 (75.5) | 124 (63.2) | 141 (75.5) | 0.012 |
| **SII** | 496 (406) | 467 (369) | 492 (412) | 0.596 |
| **LMR** | 3.58 (2.09) | 3.45 (2.02) | 3.89 (2.43) | 0.184 |

**Supplementary Table 3. Summary of complete blood count (CBC) values and derived inflammatory markers according to site of disease onset. P-values from logistic regression model.**

|  | **Total** (median, (IQR)) | **Site of onset** (median, (IQR)) | |  |
| --- | --- | --- | --- | --- |
|  | (n=1452) | **Bulbar (n=489)** | **Spinal (n=963)** | **p-value** |
| **WBC** | 6.28 (2.34) | 6.04 (2.41) | 6.4 (2.3) | 0.782 |
| **Neutrophils** | 3.77 (1.96) | 3.64 (2.05) | 3.82 (1.92) | 0.688 |
| **Lymphocytes** | 1.72 (0.8) | 1.66 (0.812) | 1.74 (0.8) | 0.800 |
| **Monocytes** | 0.48 (0.23) | 0.47 (0.23) | 0.49 (0.24) | 0.842 |
| **Platelets** | 229 (82) | 220 (88) | 233 (79.5) | 0.540 |
| **NLR** | 2.14 (1.47) | 2.12 (1.5) | 2.16 (1.47) | 0.935 |
| **PLR** | 135 (75.5) | 135 (73.8) | 134 (74.9) | 0.902 |
| **SII** | 496 (406) | 481 (402) | 507 (403) | 0.742 |
| **LMR** | 3.58 (2.09) | 3.61 (2.05) | 3.56 (2.11) | 0.932 |

**Supplementary Table 4. Summary of complete blood count (CBC) values and derived inflammatory markers according to age groups. P-values from linear regression model.**

|  | **Total** (median, (IQR)) | **Age groups** (median, (IQR)) | | |  |
| --- | --- | --- | --- | --- | --- |
|  | (n=1452) | **< 65 years (n=497)** | **65 – 75 years (n=548)** | **>75 years (n=406)** | **p-value** |
| **WBC** | 6.28 (2.34) | 6.13 (2.13) | 6.35 (2.34) | 6.38 (2.59) | 0.088 |
| **Neutrophils** | 3.77 (1.96) | 3.54 (1.7) | 3.87 (1.98) | 3.92 (2.02) | 0.0001 |
| **Lymphocytes** | 1.72 (0.8) | 1.88 (0.92) | 1.7 (0.73) | 1.54 (0.71) | 0.001 |
| **Monocytes** | 0.48 (0.23) | 0.45 (0.22) | 0.49 (0.24) | 0.5 (0.23) | 0.001 |
| **Platelets** | 229 (82) | 237 (79) | 232 (79.2) | 218 (87) | 0.095 |
| **NLR** | 2.14 (1.47) | 1.93 (1.12) | 2.25 (1.47) | 2.47 (1.77) | <0.0001 |
| **PLR** | 135 (75.5) | 126 (74.4) | 135 (73.6) | 144 (79) | 0.436 |
| **SII** | 496 (406) | 456 (326) | 516 (405) | 572 (465) | 0.001 |
| **LMR** | 3.58 (2.09) | 4.02 (2.22) | 3.5 (2.02) | 3.1 (1.67) | 0.001 |

**Supplementary Table 5. Summary of complete blood count (CBC) values and derived inflammatory markers according to sex and age groups. Linear regression models were employed to test for differences between the two sexes in trends across the age groups for cells count and inflammatory indexes. P values for sex and age interaction from the linear regression model.**

|  | **< 65 years** | | **65 – 75 years** | | **> 75 years** | |  |
| --- | --- | --- | --- | --- | --- | --- | --- |
|  | **Male (n=291)** | **Female (n=206)** | **Male (n=307)** | **Female (n=241)** | **Male (n=200)** | **Female (n=206)** | **p-value** |
| **WBC** | 6.21 (2.02) | 5.8 (2.33) | 6.58 (2.47) | 6.18 (2.1) | 6.21 (2.55) | 6.53 (2.55) | 0.0148 |
| **Neutrophils** | 3.6 (1.57) | 3.14 (1.56) | 4.03 (2.08) | 3.69 (1.96) | 3.81 (1.86) | 3.9 (2.22) | 0.0003 |
| **Lymphocytes** | 1.9 (0.872) | 1.88 (0.92) | 1.69 (0.66) | 1.75 (0.75) | 1.54 (0.64) | 1.55 (0.71) | 0.0831 |
| **Monocytes** | 0.46 (0.208) | 0.4 (0.213) | 0.51 (0.26) | 0.44 (0.237) | 0.5 (0.2) | 0.48 (0.21) | < 0.0001 |
| **Platelets** | 218 (72.5) | 254 (69) | 214 (82.5) | 244 (75.8) | 196 (70.8) | 228 (94.5) | < 0.0001 |
| **NLR** | 1.92 (1.06) | 1.78 (1.19) | 2.39 (1.53) | 1.99 (1.23) | 2.41 (1.53) | 2.47 (1.84) | < 0.0001 |
| **PLR** | 121 (57.9) | 132 (76.3) | 128 (67) | 142 (69.9) | 134 (61.7) | 147 (88.3) | 0.0001 |
| **SII** | 422 (295) | 449 (322) | 522 (398) | 488 (404) | 508 (433) | 594 (484) | 0.0125 |
| **LMR** | 3.97 (2.08) | 4.56 (2.59) | 3.16 (1.85) | 3.86 (2.35) | 3.14 (1.48) | 3.27 (2.11) | < 0.0001 |

**Supplementary Table 6. Influence of smoking status on CBC data and inflammatory markers. P values from logistic regression models.**

|  | **Active smoking** | | |
| --- | --- | --- | --- |
| *Predictors* | *OR* | *CI* | *p* |
| Neutrophil | 1.03 | 1.01 – 1.06 | **0.01** |
| Monocyte | 1 | 0.98 – 1.03 | 0.697 |
| Platelet | 1.01 | 0.98 – 1.03 | 0.65 |
| NLR | 0.99 | 0.97 – 1.01 | 0.415 |
| PLR | 0.99 | 0.97 – 1.01 | 0.233 |
| SII | 0.99 | 0.96 – 1.01 | 0.298 |
| Lymphocyte | 1.02 | 1.00 – 1.04 | 0.06 |
| LMR | 1 | 0.98 – 1.02 | 0.813 |

**Supplementary Table 7. Influence of bulbar symptoms on CBC data and inflammatory markers. P values from logistic regression models.**

|  | **Bulbar symptoms** | | |
| --- | --- | --- | --- |
| *Predictors* | *OR* | *CI* | *p* |
| Neutrophil | 1.01 | 0.98 – 1.04 | 0.688 |
| Monocyte | 0.99 | 0.92 – 1.07 | 0.842 |
| Platelet | 1.01 | 0.98 – 1.04 | 0.54 |
| NLR | 1 | 0.97 – 1.03 | 0.935 |
| PLR | 0.99 | 0.90 – 1.10 | 0.902 |
| SII | 1.01 | 0.97 – 1.04 | 0.742 |
| Lymphocyte | 1 | 0.97 – 1.02 | 0.8 |
| LMR | 1 | 0.97 – 1.03 | 0.932 |

**Supplementary Table 8. Influence of the time from symptoms onset to blood sample collection on CBC data and inflammatory markers in the whole cohort (A), according to sex (B) and age groups (< 60 years, 60-70 years, > 70 years) (C). P values from linear regression models.**

|  | 1. **Diagnostic delay** | | |
| --- | --- | --- | --- |
| *Predictors* | *β* | *CI* | *p* |
| Neutrophil | -0.14 | -4.06 – 3.78 | 0.944 |
| Monocyte | -1.24 | -5.78 – 3.30 | 0.593 |
| Platelet | 0.82 | -2.15 – 3.78 | 0.59 |
| NLR | -1.05 | -3.98 – 1.88 | 0.483 |
| PLR | -1.32 | -10.45 – 7.80 | 0.776 |
| SII | -0.5 | -3.84 – 2.84 | 0.767 |
| Lymphocyte | -0.61 | -3.09 – 1.86 | 0.627 |
| LMR | -0.38 | -2.82 – 2.06 | 0.76 |

|  | 1. **Diagnostic delay – Sex-based analysis** | | | |  | |
| --- | --- | --- | --- | --- | --- | --- |
|  | **Female (n=653)** | | | | **Male (n=799)** | |
| *Predictors* | *β* | *CI* | *p* | *β* | *CI* | *p* |
| Neutrophil | -0.67 | -4.50 – 3.17 | 0.734 | -0.18 | -4.37 – 4.01 | 0.933 |
| Monocyte | 1.92 | -8.92 – 12.76 | 0.728 | -1.82 | -6.89 – 3.26 | 0.482 |
| Platelet | 1.74 | -3.02 – 6.49 | 0.474 | 0.15 | -3.66 – 3.96 | 0.939 |
| NLR | -1.55 | -5.45 – 2.34 | 0.434 | -0.76 | -5.20 – 3.67 | 0.735 |
| PLR | -0.28 | -14.4 – 13.84 | 0.969 | -2.93 | -14.94 – 9.07 | 0.631 |
| SII | -1.74 | -6.51 – 3.03 | 0.475 | -0.1 | -4.80 – 4.60 | 0.968 |
| Lymphocyte | -0.5 | -7.70 – 6.69 | 0.891 | -0.51 | -3.19 – 2.18 | 0.712 |
| LMR | 0.15 | -5.57 – 5.88 | 0.958 | -0.38 | -3.12 – 2.35 | 0.784 |

|  | 1. **Diagnostic delay – Age-based analysis** | | | |  | |  |  |  |
| --- | --- | --- | --- | --- | --- | --- | --- | --- | --- |
|  | **< 65 years (n=497)** | | | | **65-75 years (n=548)** | | **> 75 years (n=406)** | | |
| *Predictors* | *β* | *CI* | *p* | *Β* | *CI* | *p* |  |  |  |
| Neutrophil | -1.31 | -6.98 – 4.35 | 0.649 | -0.27 | -4.42 – 3.89 | 0.9 | -0.4 | -5.92 – 5.12 | 0.886 |
| Monocyte | -4.37 | -16.36 – 7.61 | 0.474 | -0.84 | -6.48 – 4.80 | 0.77 | -1.85 | -13.7 – 10.0 | 0.759 |
| Platelet | 1.12 | -4.43 – 6.68 | 0.691 | 1.56 | -3.11 – 6.23 | 0.512 | -0.67 | -6.07 – 4.74 | 0.808 |
| NLR | 0.22 | -7.11 – 7.55 | 0.953 | -2.12 | -6.45 – 2.21 | 0.337 | -0.34 | -5.11 – 4.43 | 0.888 |
| PLR | 8.8 | -9.98 – 27.57 | 0.357 | -5.62 | -19.17 – 7.92 | 0.415 | -1.21 | -18.2 – 15.7 | 0.889 |
| SII | -0.83 | -8.37 – 6.71 | 0.829 | -0.59 | -5.43 – 4.25 | 0.811 | -0.61 | -6.66 – 5.44 | 0.842 |
| Lymphocyte | 0.03 | -7.86 – 7.93 | 0.994 | -0.62 | -3.46 – 2.23 | 0.671 | -3.65 | -13.4 – 6.04 | 0.459 |
| LMR | 1.53 | -2.77 – 5.83 | 0.484 | -1.25 | -4.59 – 2.09 | 0.462 | -1.34 | -8.94 – 6.27 | 0.729 |

**Supplementary Table 9. Summary of multivariate analysis of immune and adaptive inflammation markers in the whole cohort** (*β* estimates from linear regression; OR odds ratio from logistic regression, HR hazard ratio from Cox proportional hazard regression, 97.5% CI 97.55% confidence interval, NLR neutrophil-to-lymphocyte ratio, PLR platelet-to-lymphocyte ratio, SII systemic inflammatory index, LMR lymphocyte-to-monocyte ratio)

|  | **FVC < 75%** | | |
| --- | --- | --- | --- |
| *Predictors* | *OR* | *97.5% CI* | *P* |
| Neutrophil | 1.06 | 1.03 – 1.09 | **<0.001** |
| Monocyte | 1.01 | 0.93 – 1.09 | 0.834 |
| Platelet | 1.02 | 0.99 – 1.06 | 0.216 |
| NLR | 1.07 | 1.03 – 1.10 | **<0.001** |
| PLR | 1.15 | 1.04 – 1.28 | **0.007** |
| SII | 1.09 | 1.04 – 1.13 | **<0.001** |
| Lymphocyte | 0.97 | 0.95 – 1.00 | **0.03** |
| LMR | 1 | 0.97 – 1.02 | 0.899 |
|  | **ALSFRS-R PROGRESSION RATE** | | |
| *Predictors* | *β* | *97.5% CI* | *P* |
| Neutrophil | 1.03 | 1.01 – 1.05 | **0.001** |
| Monocyte | 1.05 | 1.00 – 1.10 | **0.041** |
| Platelet | 1 | 0.98 – 1.02 | 0.801 |
| NLR | 1.03 | 1.01 – 1.05 | **0.008** |
| PLR | 1.05 | 0.99 – 1.11 | 0.136 |
| SII | 1.03 | 1.01 – 1.05 | **0.006** |
| Lymphocyte | 0.99 | 0.98 – 1.01 | 0.413 |
| LMR | 0.99 | 0.98 – 1.01 | 0.263 |
|  | **COGNITIVE IMPAIRMENT** | | |
| *Predictors* | *OR* | *97.5% CI* | *P* |
| Neutrophil | 1.01 | 0.95 – 1.07 | 0.724 |
| Monocyte | 0.84 | 0.73 – 0.97 | **0.017** |
| Platelet | 1 | 0.94 – 1.07 | 0.975 |
| NLR | 1.05 | 0.98 – 1.12 | 0.171 |
| PLR | 1.16 | 0.94 – 1.44 | 0.167 |
| SII | 1.07 | 0.99 – 1.16 | 0.093 |
| Lymphocyte | 0.97 | 0.93 – 1.02 | 0.225 |
| LMR | 1.03 | 0.99 – 1.08 | 0.166 |
|  | **FTD** | | |
| *Predictors* | *OR* | *97.5% CI* | *P* |
| Neutrophil | 1 | 0.97 – 1.03 | 0.928 |
| Monocyte | 0.92 | 0.86 – 0.99 | **0.036** |
| Platelet | 1 | 0.97 – 1.03 | 0.985 |
| NLR | 1.03 | 0.99 – 1.06 | 0.134 |
| PLR | 1.11 | 0.99 – 1.24 | 0.068 |
| SII | 1.03 | 0.99 – 1.07 | 0.138 |
| Lymphocyte | 0.99 | 0.97 – 1.01 | 0.259 |
| LMR | 1.02 | 0.99 – 1.04 | 0.138 |
|  | **SURVIVAL** | | |
| *Predictors* | *HR* | *97.5% CI* | *P* |
| Neutrophil | 1.11 | 1.05 – 1.17 | **<0.001** |
| Monocyte | 1.02 | 0.97 – 1.07 | 0.366 |
| Platelet | 0.99 | 0.93 – 1.04 | 0.616 |
| NLR | 1.13 | 1.07 – 1.18 | **<0.001** |
| PLR | 1.01 | 0.96 – 1.07 | 0.67 |
| SII | 1.12 | 1.06 – 1.17 | **<0.001** |
| Lymphocyte | 0.96 | 0.88 – 1.05 | 0.419 |
| LMR | 0.95 | 0.89 – 1.02 | 0.14 |
|  | **KING’S STAGING** | | |
| *Predictors* | *β* | *97.5% CI* | *P* |
| Neutrophil | **0.06** | **0.01 – 0.11** | **0.010** |
| Monocyte | 0.02 | -0.06 – 0.09 | 0.692 |
| Platelet | 0.03 | -0.02 – 0.08 | 0.230 |
| NLR | **0.07** | **0.02 – 0.12** | **0.006** |
| PLR | **0.24** | **0.09 – 0.40** | **0.002** |
| SII | **0.11** | **0.05 – 0.16** | **<0.001** |
| Lymphocyte | -0.04 | -0.08 – 0.00 | 0.081 |
| LMR | -0.01 | -0.05 – 0.03 | 0.562 |

**Supplementary Table 10. Summary of multivariate analysis of immune and adaptive inflammation markers according to sex** (*β* estimates from linear regression; OR odds ratio from logistic regression, HR hazard ratio from Cox proportional hazard regression, 97.5% CI 97.55% confidence interval, NLR neutrophil-to-lymphocyte ratio, PLR platelet-to-lymphocyte ratio, SII systemic inflammatory index, LMR lymphocyte-to-monocyte ratio)

|  |  | **FVC < 75%** | | |  |  |
| --- | --- | --- | --- | --- | --- | --- |
|  | **Female (n=653)** | | | **Male (n=799)** | | |
| *Predictors* | *OR* | *97.5% CI* | *P* | *Estimates* | *97.5% CI* | *P* |
| Neutrophil | 1.05 | 1.00 – 1.10 | **0.036** | 1.07 | 1.02 – 1.12 | **0.005** |
| Monocyte | 0.97 | 0.85 – 1.11 | 0.641 | 1.03 | 0.93 – 1.13 | 0.573 |
| Platelet | 1.03 | 0.98 – 1.09 | 0.217 | 1.01 | 0.97 – 1.06 | 0.594 |
| NLR | 1.08 | 1.03 – 1.13 | **0.001** | 1.05 | 1.00 – 1.10 | **0.033** |
| PLR | 1.35 | 1.15 – 1.59 | **<0.001** | 1.05 | 0.92 – 1.19 | 0.488 |
| SII | 1.14 | 1.07 – 1.21 | **<0.001** | 1.06 | 1.00 – 1.12 | **0.039** |
| Lymphocyte | 0.91 | 0.83 – 0.98 | **0.018** | 0.99 | 0.96 – 1.02 | 0.438 |
| LMR | 0.97 | 0.91 – 1.04 | 0.36 | 1 | 0.98 – 1.03 | 0.8 |
|  |  | **ALSFRS-R PROGRESSION RATE** | | |  |  |
|  | **Female (n=653)** | | | **Male (n=799)** | | |
| *Predictors* | *β* | *97.5% CI* | *P* | *Estimates* | *97.5% CI* | *P* |
| Neutrophil | 1.03 | 1.01 – 1.05 | **0.012** | 1.05 | 1.01 – 1.08 | **0.004** |
| Monocyte | 1.09 | 1.02 – 1.16 | **0.009** | 1.06 | 0.99 – 1.12 | 0.083 |
| Platelet | 0.99 | 0.97 – 1.02 | 0.688 | 1 | 0.97 – 1.03 | 0.955 |
| NLR | 1.03 | 1.00 – 1.05 | **0.02** | 1.04 | 1.01 – 1.08 | **0.011** |
| PLR | 1 | 0.97 – 1.03 | 0.851 | 1.06 | 0.98 – 1.15 | 0.173 |
| SII | 1.04 | 1.01 – 1.06 | **0.007** | 1.03 | 1.00 – 1.07 | **0.039** |
| Lymphocyte | 0.97 | 0.93 – 1.01 | 0.129 | 0.99 | 0.97 – 1.01 | 0.415 |
| LMR | 0.97 | 0.94 – 1.00 | **0.049** | 0.99 | 0.97 – 1.01 | 0.413 |
|  |  | **COGNITIVE IMPAIRMENT** | | |  |  |
|  | **Female (n=653)** | | | **Male (n=799)** | | |
| *Predictors* | *OR* | *97.5% CI* | *P* | *Estimates* | *97.5% CI* | *P* |
| Neutrophil | 0.98 | 0.94 – 1.02 | 0.291 | 1.03 | 0.98 – 1.08 | 0.209 |
| Monocyte | 0.87 | 0.77 – 0.98 | **0.023** | 0.96 | 0.88 – 1.05 | 0.388 |
| Platelet | 0.99 | 0.94 – 1.05 | 0.783 | 1 | 0.96 – 1.04 | 0.981 |
| NLR | 1.02 | 0.97 – 1.07 | 0.502 | 1.04 | 0.99 – 1.09 | 0.145 |
| PLR | 1.19 | 1.01 – 1.40 | **0.036** | 1.02 | 0.88 – 1.19 | 0.774 |
| SII | 1.02 | 0.97 – 1.08 | 0.451 | 1.04 | 0.98 – 1.10 | 0.245 |
| Lymphocyte | 0.91 | 0.85 – 0.99 | **0.022** | 0.99 | 0.97 – 1.02 | 0.599 |
| LMR | 1.02 | 0.96 – 1.08 | 0.548 | 1.02 | 0.99 – 1.04 | 0.184 |
|  |  | **FTD** | | |  |  |
|  | **Female (n=653)** | | | **Male (n=799)** | | |
| *Predictors* | *OR* | *97.5% CI* | *P* | *Estimates* | *97.5% CI* | *P* |
| Neutrophil | 0.97 | 0.90 – 1.05 | 0.461 | 1.06 | 0.97 – 1.16 | 0.201 |
| Monocyte | 0.79 | 0.63 – 1.00 | **0.046** | 0.88 | 0.73 – 1.05 | 0.146 |
| Platelet | 1.01 | 0.92 – 1.12 | 0.778 | 0.99 | 0.91 – 1.07 | 0.754 |
| NLR | 1.03 | 0.94 – 1.12 | 0.591 | 1.07 | 0.97 – 1.19 | 0.168 |
| PLR | 1.28 | 0.95 – 1.73 | 0.106 | 1.02 | 0.76 – 1.38 | 0.885 |
| SII | 1.05 | 0.95 – 1.17 | 0.345 | 1.08 | 0.96 – 1.22 | 0.201 |
| Lymphocyte | 0.89 | 0.77 – 1.02 | 0.096 | 0.98 | 0.94 – 1.03 | 0.432 |
| LMR | 1.07 | 0.95 – 1.20 | 0.24 | 1.02 | 0.98 – 1.07 | 0.312 |
|  |  | **SURVIVAL** | | |  |  |
|  | **Female (n=653)** | | | **Male (n=799)** | | |
| *Predictors* | *HR* | *97.5% CI* | *P* | *Estimates* | *97.5% CI* | *P* |
| Neutrophil | 1.09 | 1.01 – 1.19 | **0.03** | 1.07 | 0.97 – 1.17 | 0.165 |
| Monocyte | 1.17 | 0.92 – 1.49 | 0.197 | 0.98 | 0.79 – 1.20 | 0.815 |
| Platelet | 1.08 | 0.97 – 1.20 | 0.154 | 0.95 | 0.86 – 1.03 | 0.22 |
| NLR | 1.15 | 1.07 – 1.25 | **<0.001** | 1.11 | 1.01 – 1.22 | **0.028** |
| PLR | 1.04 | 0.95 – 1.13 | 0.392 | 1.08 | 0.84 – 1.39 | 0.558 |
| SII | 1.3 | 1.18 – 1.44 | **<0.001** | 1.07 | 0.97 – 1.18 | 0.156 |
| Lymphocyte | 0.84 | 0.70 – 1.00 | **0.045** | 1 | 0.91 – 1.09 | 0.991 |
| LMR | 0.83 | 0.72 – 0.96 | **0.013** | 1.02 | 0.95 – 1.08 | 0.639 |
|  |  | **KING’S STAGING** | | |  |  |
|  | **Female (n=653)** | | | **Male (n=799)** | | |
| *Predictors* | *β* | *97.5% CI* | *P* | *Estimates* | *97.5% CI* | *P* |
| Neutrophil | 0.01 | -0.06 – 0.08 | 0.715 | **0.12** | **0.05 – 0.19** | **0.001** |
| Monocyte | 0.05 | -0.14 – 0.25 | 0.582 | 0.01 | -0.07 – 0.10 | 0.802 |
| Platelet | -0.01 | -0.09 – 0.08 | 0.862 | 0.05 | -0.01 – 0.12 | 0.092 |
| NLR | 0.03 | -0.04 – 0.10 | 0.417 | **0.11** | **0.04 – 0.19** | **0.002** |
| PLR | 0.22 | -0.03 – 0.46 | 0.085 | **0.25** | **0.05 – 0.45** | **0.016** |
| SII | 0.06 | -0.02 – 0.14 | 0.153 | **0.15** | **0.07 – 0.23** | **<0.001** |
| Lymphocyte | -0.11 | -0.24 – 0.02 | 0.089 | -0.03 | -0.07 – 0.02 | 0.244 |
| LMR | -0.04 | -0.15 – 0.06 | 0.397 | 0 | -0.05 – 0.04 | 0.908 |

**Supplementary Table 11. Summary of multivariate analysis of immune and adaptive inflammation markers according to age group (< 60 years, 60-70 years, > 70 years)** (*β* estimates from linear regression; OR odds ratio from logistic regression, HR hazard ratio from Cox proportional hazard regression, 97.5% CI 97.55% confidence interval, NLR neutrophil-to-lymphocyte ratio, PLR platelet-to-lymphocyte ratio, SII systemic inflammatory index, LMR lymphocyte-to-monocyte ratio)

|  | **FVC < 75%** | | | | | | | | |
| --- | --- | --- | --- | --- | --- | --- | --- | --- | --- |
|  | **< 65 years (n=497)** | | | **65-75 years (n=548)** | | | **> 75 years (n=406)** | | |
| *Predictors* | *OR* | *97.5% CI* | *P* | *Estimates* | *97.5% CI* | *P* | *Estimates* | *97.5% CI* | *p* |
| Neutrophil | 1.06 | 1.00 – 1.12 | 0.066 | 1.05 | 1.01 – 1.10 | **0.026** | 1.08 | 1.00 – 1.18 | 0.052 |
| Monocyte | 1.01 | 0.89 – 1.15 | 0.877 | 0.97 | 0.86 – 1.09 | 0.619 | 1.12 | 0.93 – 1.34 | 0.236 |
| Platelet | 1.02 | 0.96 – 1.08 | 0.487 | 1.03 | 0.98 – 1.08 | 0.269 | 1.02 | 0.94 – 1.11 | 0.649 |
| NLR | 1.06 | 0.98 – 1.14 | 0.125 | 1.06 | 1.01 – 1.11 | **0.021** | 1.08 | 1.01 – 1.16 | **0.019** |
| PLR | 1.15 | 0.95 – 1.39 | 0.165 | 1.12 | 0.97 – 1.29 | 0.117 | 1.29 | 1.01 – 1.64 | **0.043** |
| SII | 1.09 | 1.00 – 1.17 | **0.038** | 1.07 | 1.01 – 1.13 | **0.013** | 1.15 | 1.04 – 1.27 | **0.005** |
| Lymphocyte | 0.97 | 0.89 – 1.05 | 0.395 | 0.99 | 0.96 – 1.02 | 0.465 | 0.89 | 0.78 – 1.01 | 0.065 |
| LMR | 1.01 | 0.97 – 1.06 | 0.522 | 1 | 0.97 – 1.04 | 0.903 | 0.94 | 0.85 – 1.03 | 0.19 |
|  | **ALSFRS-R PROGRESSION RATE** | | | | | | | | |
|  | **< 65 years (n=497)** | | | **65-75 years (n=548)** | | | **> 75 years (n=406)** | | |
| *Predictors* | *β* | *97.5% CI* | *p* | *Estimates* | *97.5% CI* | *P* | *Estimates* | *97.5% CI* | *p* |
| Neutrophil | 1.01 | 0.96 – 1.05 | 0.774 | 1.05 | 1.02 – 1.07 | **<0.001** | 1.03 | 0.98 – 1.07 | 0.228 |
| Monocyte | 1.01 | 0.92 – 1.10 | 0.886 | 1.09 | 1.03 – 1.16 | **0.005** | 1.03 | 0.94 – 1.13 | 0.507 |
| Platelet | 0.97 | 0.93 – 1.01 | 0.089 | 1.02 | 0.99 – 1.05 | 0.135 | 1.01 | 0.97 – 1.05 | 0.62 |
| NLR | 1.02 | 0.97 – 1.08 | 0.488 | 1.04 | 1.02 – 1.07 | **<0.001** | 1 | 0.96 – 1.04 | 0.977 |
| PLR | 1.01 | 0.88 – 1.15 | 0.933 | 1.08 | 1.01 – 1.16 | **0.026** | 1.01 | 0.89 – 1.14 | 0.905 |
| SII | 1.01 | 0.95 – 1.06 | 0.833 | 1.05 | 1.02 – 1.07 | **<0.001** | 1.01 | 0.97 – 1.06 | 0.529 |
| Lymphocyte | 0.96 | 0.91 – 1.02 | 0.196 | 0.99 | 0.98 – 1.01 | 0.308 | 1.07 | 0.99 – 1.15 | 0.102 |
| LMR | 0.99 | 0.96 – 1.03 | 0.679 | 0.99 | 0.97 – 1.00 | 0.139 | 1.02 | 0.96 – 1.08 | 0.502 |
|  | **COGNITIVE IMPAIRMENT** | | | | | | | | |
|  | **< 65 years (n=497)** | | | **65-75 years (n=548)** | | | **> 75 years (n=406)** | | |
| *Predictors* | *OR* | *CI* | *p* | *Estimates* | *97.5% CI* | *P* | *Estimates* | *97.5% CI* | *p* |
| Neutrophil | 1.05 | 0.94 – 1.18 | 0.366 | 1 | 0.93 – 1.08 | 0.991 | 1.03 | 0.91 – 1.17 | 0.646 |
| Monocyte | 0.79 | 0.63 – 0.99 | **0.041** | 0.84 | 0.67 – 1.04 | 0.105 | 0.85 | 0.61 – 1.20 | 0.35 |
| Platelet | 1.03 | 0.92 – 1.16 | 0.584 | 0.99 | 0.90 – 1.08 | 0.804 | 1 | 0.85 – 1.18 | 0.98 |
| NLR | 1.05 | 0.96 – 1.15 | 0.316 | 1.01 | 0.93 – 1.10 | 0.756 | 1.21 | 0.98 – 1.50 | 0.073 |
| PLR | 1.11 | 0.76 – 1.62 | 0.577 | 1.12 | 0.83 – 1.53 | 0.455 | 1.49 | 0.92 – 2.43 | 0.104 |
| SII | 1.13 | 0.96 – 1.33 | 0.143 | 1.04 | 0.95 – 1.15 | 0.394 | 1.16 | 0.96 – 1.41 | 0.122 |
| Lymphocyte | 0.99 | 0.86 – 1.14 | 0.874 | 0.98 | 0.93 – 1.03 | 0.39 | 0.7 | 0.54 – 0.90 | **0.006** |
| LMR | 1.11 | 1.03 – 1.19 | **0.006** | 1.01 | 0.96 – 1.07 | 0.657 | 0.85 | 0.66 – 1.10 | 0.205 |
|  | **FTD** | | | | | | | | |
|  | **< 65 years (n=497)** | | | **65-75 years (n=548)** | | | **> 75 years (n=406)** | | |
| *Predictors* | *OR* | *97.5% CI* | *p* | *Estimates* | *97.5% CI* | *P* | *Estimates* | *97.5% CI* | *p* |
| Neutrophil | 1.03 | 0.97 – 1.09 | 0.366 | 0.99 | 0.96 – 1.03 | 0.73 | 1.03 | 0.96 – 1.11 | 0.377 |
| Monocyte | 0.9 | 0.81 – 1.01 | 0.062 | 0.93 | 0.83 – 1.04 | 0.219 | 0.91 | 0.75 – 1.11 | 0.357 |
| Platelet | 1.03 | 0.98 – 1.09 | 0.253 | 0.99 | 0.94 – 1.04 | 0.637 | 1 | 0.91 – 1.10 | 0.947 |
| NLR | 1.01 | 0.97 – 1.06 | 0.561 | 1.01 | 0.97 – 1.06 | 0.6 | 1.15 | 1.02 – 1.30 | **0.021** |
| PLR | 1.07 | 0.89 – 1.29 | 0.464 | 1.1 | 0.94 – 1.28 | 0.256 | 1.31 | 0.99 – 1.74 | 0.057 |
| SII | 1.06 | 0.98 – 1.15 | 0.163 | 1.01 | 0.96 – 1.07 | 0.645 | 1.13 | 1.01 – 1.26 | **0.03** |
| Lymphocyte | 0.99 | 0.93 – 1.06 | 0.801 | 0.99 | 0.97 – 1.02 | 0.536 | 0.82 | 0.71 – 0.94 | **0.006** |
| LMR | 1.05 | 1.01 – 1.09 | **0.006** | 1.01 | 0.98 – 1.04 | 0.515 | 0.93 | 0.80 – 1.07 | 0.302 |
|  | **SURVIVAL** | | | | | | | | |
|  | **< 65 years (n=497)** | | | **65-75 years (n=548)** | | | **> 75 years (n=406)** | | |
| *Predictors* | *HR* | *97.5% CI* | *p* | *Estimates* | *97.5% CI* | *P* | *Estimates* | *97.5% CI* | *P* |
| Neutrophil | 1.02 | 0.91 – 1.15 | 0.678 | 1.08 | 1.00 – 1.17 | **0.04** | 1.16 | 1.05 – 1.29 | **0.005** |
| Monocyte | 0.94 | 0.74 – 1.20 | 0.628 | 1.02 | 0.93 – 1.12 | 0.657 | 1 | 0.94 – 1.06 | 0.984 |
| Platelet | 1.01 | 0.91 – 1.13 | 0.852 | 0.98 | 0.90 – 1.08 | 0.735 | 1.01 | 0.91 – 1.12 | 0.842 |
| NLR | 1.07 | 0.96 – 1.21 | 0.234 | 1.13 | 1.05 – 1.21 | **0.001** | 1.1 | 1.00 – 1.21 | **0.047** |
| PLR | 1.03 | 0.93 – 1.15 | 0.563 | 1 | 0.92 – 1.07 | 0.914 | 1.21 | 0.86 – 1.70 | 0.273 |
| SII | 1.1 | 0.98 – 1.22 | 0.094 | 1.09 | 1.01 – 1.17 | **0.022** | 1.19 | 1.06 – 1.32 | **0.002** |
| Lymphocyte | 0.92 | 0.77 – 1.09 | 0.331 | 1.02 | 0.95 – 1.10 | 0.581 | 0.99 | 0.79 – 1.24 | 0.936 |
| LMR | 1 | 0.91 – 1.10 | 0.966 | 0.97 | 0.88 – 1.07 | 0.545 | 1.02 | 0.85 – 1.24 | 0.804 |
|  | **KING’S STAGING** | | | | | | | | |
|  | **< 65 years (n=497)** | | | **65-75 years (n=548)** | | | **> 75 years (n=406)** | | |
| *Predictors* | *HR* | *97.5% CI* | *p* | *Estimates* | *97.5% CI* | *P* | *Estimates* | *97.5% CI* | *P* |
| Neutrophil | 0.03 | -0.06 – 0.13 | 0.456 | **0.11** | **0.04 – 0.17** | **0.002** | -0.01 | -0.10 – 0.09 | 0.877 |
| Monocyte | 0.1 | -0.09 – 0.29 | 0.323 | 0 | -0.09 – 0.09 | 0.999 | -0.04 | -0.26 – 0.19 | 0.753 |
| Platelet | 0 | -0.09 – 0.09 | 0.943 | 0.07 | -0.00 – 0.15 | 0.063 | -0.01 | -0.12 – 0.09 | 0.784 |
| NLR | -0.02 | -0.11 – 0.07 | 0.679 | **0.11** | **0.04 – 0.18** | **0.002** | 0.03 | -0.07 – 0.12 | 0.552 |
| PLR | 0.08 | -0.22 – 0.38 | 0.596 | 0.01 | -0.08 – 0.11 | 0.772 | 0.29 | -0.03 – 0.62 | 0.079 |
| SII | 0.05 | -0.08 – 0.17 | 0.458 | **0.15** | **0.07 – 0.22** | **<0.001** | 0.06 | -0.05 – 0.17 | 0.315 |
| Lymphocyte | -0.02 | -0.15 – 0.10 | 0.715 | -0.03 | -0.08 – 0.01 | 0.149 | -0.18 | -0.37 – 0.02 | 0.079 |
| LMR | 0.05 | -0.02 – 0.12 | 0.167 | -0.03 | -0.09 – 0.02 | 0.215 | -0.04 | -0.20 – 0.11 | 0.58 |

**Supplementary Table 12. Interaction analysis of inflammatory markers, age, and sex.** Coef, coefficient of age, sex and inflammation interaction. P, p-vlaue of the interaction parameter in the logistic mixed-regression model (FVC, FTD), linear regression model (ALSFRS-R PROGRESSION RATE) or the Cox-regression (SURVIVAL).

| **Outcome** | **Parameter** | **Sex** | **Coef** | ***2.5% CI*** | ***97.5% CI*** | **P** |  |
| --- | --- | --- | --- | --- | --- | --- | --- |
|  |  |  |  |  |  |  |  |
| **FVC** | Lymphocyte | Female | 0.998278 | 0.997066 | 0.999492 | **0.005501** |  |
|  |  | Male | 0.999772 | 0.999345 | 1.000199 | 0.294062 |  |
|  | Monocyte | Female | 1.000508 | 0.998623 | 1.002396 | 0.597391 |  |
|  |  | Male | 1.000583 | 0.999155 | 1.002012 | 0.423597 |  |
|  | Neutrophil | Female | 1.000871 | 1.000211 | 1.001532 | **0.009796** |  |
|  |  | Male | 1.001054 | 1.000363 | 1.001746 | **0.002837** |  |
|  | Platelet | Female | 1.000363 | 0.999597 | 1.001131 | 0.352586 |  |
|  |  | Male | 0.999923 | 0.999286 | 1.00056 | 0.81194 |  |
|  | PLR | Female | 1.004414 | 1.002112 | 1.006722 | **0.000177** |  |
|  |  | Male | 1.00038 | 0.998529 | 1.002234 | 0.687567 |  |
|  | SII | Female | 1.001939 | 1.001051 | 1.002828 | **<0.0001** |  |
|  |  | Male | 1.000878 | 1.000108 | 1.001649 | 0.025456 |  |
|  | LMR | Female | 0.999157 | 0.998191 | 1.000125 | 0.087668 |  |
|  |  | Male | 0.99999 | 0.999558 | 1.000423 | 0.964778 |  |
|  | NLR | Female | 1.001193 | 1.000536 | 1.00185 | **0.000382** |  |
|  |  | Male | 1.000897 | 1.000209 | 1.001586 | 0.010617 |  |
| **ALSFRS-R PROGRESSION RATE** | Lymphocyte | Female | 0.999581 | 0.998829 | 1.000334 | 0.2759 |  |
|  |  | Male | 0.999905 | 0.999643 | 1.000168 | 0.4791 |  |
|  | Monocyte | Female | 1.000898 | 0.999844 | 1.001954 | 0.0954 |  |
|  |  | Male | 1.000141 | 0.999673 | 1.000608 | 0.5559 |  |
|  | Neutrophil | Female | 1.000356 | 0.999992 | 1.00072 | 0.0553 |  |
|  |  | Male | 1.000643 | 1.000252 | 1.001034 | 0.0013 |  |
|  | Platelet | Female | 1.000066 | 0.999603 | 1.000529 | 0.781 |  |
|  |  | Male | 1.000013 | 0.999671 | 1.000355 | 0.941 |  |
|  | PLR | Female | 1.000952 | 0.999693 | 1.002212 | 0.139 |  |
|  |  | Male | 1.000579 | 0.999532 | 1.001626 | 0.279 |  |
|  | SII | Female | 1.000468 | 1.000059 | 1.000877 | 0.0251 |  |
|  |  | Male | 1.000471 | 1.000059 | 1.000882 | 0.0251 |  |
|  | LMR | Female | 0.999385 | 0.998817 | 0.999953 | 0.0341 |  |
|  |  | Male | 0.999918 | 0.999654 | 1.000183 | 0.5447 |  |
|  | NLR | Female | 1.000291 | 0.999942 | 1.000641 | 0.10288 |  |
|  |  | Male | 1.000552 | 1.000145 | 1.00096 | 0.00797 |  |
| **FTD** | Lymphocyte | Female | 0.998351 | 0.997238 | 0.999459 | 0.003672 |  |
|  |  | Male | 0.999901 | 0.999552 | 1.000249 | 0.576793 |  |
|  | Monocyte | Female | 0.998549 | 0.9968 | 1.000301 | 0.104873 |  |
|  |  | Male | 0.999735 | 0.999084 | 1.000385 | 0.424084 |  |
|  | Neutrophil | Female | 0.999774 | 0.999202 | 1.000345 | 0.437825 |  |
|  |  | Male | 1.000303 | 0.999634 | 1.000973 | 0.374915 |  |
|  | Platelet | Female | 0.999787 | 0.999013 | 1.000561 | 0.589888 |  |
|  |  | Male | 1.000001 | 0.999386 | 1.000617 | 0.99675 |  |
|  | PLR | Female | 1.002792 | 1.000633 | 1.004956 | 0.011455\ |  |
|  |  | Male | 1.000259 | 0.998152 | 1.002372 | 0.809616 |  |
|  | SII | Female | 1.000455 | 0.999706 | 1.001204 | 0.234376 |  |
|  |  | Male | 1.000387 | 0.999475 | 1.001301 | 0.40589 |  |
|  | LMR | Female | 0.999886 | 0.998986 | 1.000787 | 0.805 |  |
|  |  | Male | 1.000254 | 0.999883 | 1.000625 | 0.18 |  |
|  | NLR | Female | 1.000383 | 0.999723 | 1.001043 | 0.255648 |  |
|  |  | Male | 1.000476 | 0.999692 | 1.001261 | 0.234571 |  |
|  | **Parameter** | **Sex** | **OR** | ***2.5% CI*** | ***97.5% CI*** | **P** |  |
| **SURVIVAL** | Lymphocyte | Female | 0.997645 | 0.995117 | 1.000179 | 0.0685 |  |
|  |  | Male | 1.000157 | 0.998844 | 1.001471 | 0.8151 |  |
|  | Monocyte | Female | 1.002875 | 0.999499 | 1.006261 | 0.0952 |  |
|  |  | Male | 0.999572 | 0.996465 | 1.002689 | 0.7875 |  |
|  | Neutrophil | Female | 1.001473 | 1.000378 | 1.002569 | **0.00837** |  |
|  |  | Male | 1.000996 | 0.999599 | 1.002395 | 0.1625 |  |
|  | Platelet | Female | 1.001055 | 0.999506 | 1.002607 | 0.182 |  |
|  |  | Male | 0.998942 | 0.997608 | 1.000278 | 0.121 |  |
|  | PLR | Female | 1.000415 | 0.999232 | 1.0016 | 0.492 |  |
|  |  | Male | 1.000135 | 0.99623 | 1.004056 | 0.946 |  |
|  | SII | Female | 1.003174 | 1.001884 | 1.004465 | **<0.0001** |  |
|  |  | Male | 1.001012 | 0.999566 | 1.00246 | 0.17 |  |
|  | LMR | Female | 0.997237 | 0.99517 | 0.999308 | **0.00895** |  |
|  |  | Male | 1.000321 | 0.999366 | 1.001276 | 0.51037 |  |
|  | NLR | Female | 1.001796 | 1.00077 | 1.002822 | **0.000598** |  |
|  |  | Male | 1.001557 | 1.000119 | 1.002996 | 0.033752 |  |

**Supplementary Table 13. Summary of sensitivity analysis performed on ALSFRS-R score at diagnosis in the whole cohort (A), according to sex (B) and age groups (< 60 years, 60-70 years, > 70 years) (C)** (*β* estimates from linear regression; 97.5% CI 97.55% confidence interval, NLR neutrophil-to-lymphocyte ratio, PLR platelet-to-lymphocyte ratio, SII systemic inflammatory index, LMR lymphocyte-to-monocyte ratio)

| 1. **ALSFRS-R SCORE AT DIAGNOSIS** | | | |
| --- | --- | --- | --- |
| *Predictors* | *Β* | *97.5% CI* | *P* |
| Neutrophil | **1.07** | **1.02 – 1.12** | **0.010** |
| Monocyte | 1.02 | 0.94 – 1.10 | 0.692 |
| Platelet | 1.03 | 0.98 – 1.08 | 0.230 |
| NLR | **1.07** | **1.02 – 1.13** | **0.006** |
| PLR | **1.27** | **1.09 – 1.49** | **0.002** |
| SII | **1.11** | **1.05 – 1.18** | **<0.001** |
| Lymphocyte | 0.96 | 0.92 – 1.00 | 0.081 |
| LMR | 0.99 | 0.95 – 1.03 | 0.562 |

| 1. **ALSFRS-R SCORE AT DIAGNOSIS – SEX-BASED ANALYSIS** | | | | | | |
| --- | --- | --- | --- | --- | --- | --- |
|  | **Female (n=653)** | | | **Male (n=799)** | | |
| *Predictors* | *Β* | *97.5% CI* | *P* | *Estimates* | *97.5% CI* | *P* |
| Neutrophil | 1.01 | 0.95 – 1.08 | 0.715 | **1.12** | **1.05 – 1.20** | **0.001** |
| Monocyte | 1.06 | 0.87 – 1.28 | 0.582 | 1.01 | 0.93 – 1.10 | 0.802 |
| Platelet | 0.99 | 0.91 – 1.08 | 0.862 | 1.06 | 0.99 – 1.13 | 0.092 |
| NLR | 1.03 | 0.96 – 1.10 | 0.417 | **1.12** | **1.04 – 1.21** | **0.002** |
| PLR | 1.24 | 0.97 – 1.58 | **0.085** | **1.28** | **1.05 – 1.56** | **0.016** |
| SII | 1.06 | 0.98 – 1.15 | 0.153 | **1.16** | **1.07 – 1.25** | **<0.001** |
| Lymphocyte | 0.89 | 0.79 – 1.02 | 0.089 | 0.97 | 0.93 – 1.02 | 0.244 |
| LMR | 0.96 | 0.86 – 1.06 | 0.397 | 1 | 0.95 – 1.04 | 0.908 |

| 1. **ALSFRS-R SCORE AT DIAGNOSIS – AGE-BASED ANALYSIS** | | | | | | | | | |
| --- | --- | --- | --- | --- | --- | --- | --- | --- | --- |
|  | **< 65 years (n=497)** | | | **65-75 years (n=548)** | | | **> 75 years (n=406)** | | |
| *Predictors* | *β* | *97.5% CI* | *p* | *Estimates* | *97.5% CI* | *p* | *Estimates* | *97.5% CI* | *P* |
| Neutrophil | 1.04 | 0.95 – 1.13 | 0.456 | **1.11** | **1.04 – 1.19** | **0.002** | 0.99 | 0.90 – 1.09 | 0.877 |
| Monocyte | 1.1 | 0.91 – 1.33 | 0.323 | 1 | 0.91 – 1.10 | 0.999 | 0.96 | 0.77 – 1.21 | 0.753 |
| Platelet | 1 | 0.91 – 1.09 | 0.943 | 1.07 | 1.00 – 1.16 | 0.063 | 0.99 | 0.89 – 1.09 | 0.784 |
| NLR | 0.98 | 0.90 – 1.07 | 0.679 | **1.12** | **1.04 – 1.20** | **0.002** | 1.03 | 0.94 – 1.13 | 0.552 |
| PLR | 1.08 | 0.80 – 1.47 | 0.596 | 1.01 | 0.92 – 1.11 | 0.772 | 1.34 | 0.97 – 1.86 | 0.079 |
| SII | 1.05 | 0.93 – 1.18 | 0.458 | **1.16** | **1.07 – 1.25** | **<0.001** | 1.06 | 0.95 – 1.18 | 0.315 |
| Lymphocyte | 0.98 | 0.86 – 1.11 | 0.715 | 0.97 | 0.92 – 1.01 | 0.149 | 0.84 | 0.69 – 1.02 | 0.079 |
| LMR | 1.05 | 0.98 – 1.12 | 0.167 | 0.97 | 0.91 – 1.02 | 0.215 | 0.96 | 0.82 – 1.12 | 0.58 |
